# Allometric versus traditional body-shape indices and risk of colorectal cancer: a Mendelian randomization analysis

**DOI:** 10.1038/s41366-024-01479-6

**Published:** 2024-01-31

**Authors:** Marina O. Rontogianni, Emmanouil Bouras, Elom Kouassivi Aglago, Heinz Freisling, Neil Murphy, Michelle Cotterchio, Jochen Hampe, Annika Lindblom, Rish K. Pai, Paul D. P. Pharoah, Amanda I. Phipps, Franzel J. B. van Duijnhoven, Kala Visvanathan, Bethany van Guelpen, Christopher I. Li, Hermann Brenner, Andrew J. Pellatt, Shuji Ogino, Marc J. Gunter, Ulrike Peters, Sofia Christakoudi, Konstantinos K. Tsilidis

**Affiliations:** 1 Department of Hygiene and Epidemiology, University of Ioannina School of Medicine, Ioannina, Greece; 2 Department of Hygiene, Social-Preventive Medicine and Medical Statistics, Aristotle University of Thessaloniki School of Medicine, Thessaloniki, Greece; 3 Department of Epidemiology and Biostatistics, School of Public Health, Imperial College London, Norfolk Place, London, UK; 4 Nutrition and Metabolism Branch, International Agency for Research on Cancer (IARC-WHO), Lyon, France; 5 Ontario Health (Cancer Care Ontario), Toronto, ON Canada; 6 Dalla Lana School of Public Health, University of Toronto, Toronto, ON Canada; 7 Department of Medicine I, University Hospital Dresden, Technische Universität Dresden (TU Dresden), Dresden, Germany; 8 Department of Clinical Genetics, Karolinska University Hospital, Stockholm, Sweden; 9 Department of Molecular Medicine and Surgery, Karolinska Institutet, Stockholm, Sweden; 10 Department of Laboratory Medicine and Pathology, Mayo Clinic Arizona, Scottsdale, AZ USA; 11 Department of Public Health and Primary Care, University of Cambridge, Cambridge, UK; 12 Public Health Sciences Division, Fred Hutchinson Cancer Research Center, Seattle, WA USA; 13 Department of Epidemiology, University of Washington School of Public Health, Seattle, WA USA; 14 Division of Human Nutrition and Health, Wageningen University & Research, Wageningen, The Netherlands; 15 Department of Epidemiology, Johns Hopkins Bloomberg School of Public Health, Baltimore, MD USA; 16 Department of Radiation Sciences, Oncology, Umeå University, Umeå, Sweden; 17 Wallenberg Centre for Molecular Medicine, Umeå University, Umeå, Sweden; 18 Translational Research Program and Epidemiology Program, Fred Hutchinson Cancer Center, Seattle, WA USA; 19 Division of Clinical Epidemiology and Aging Research, German Cancer Research Center (DKFZ), Heidelberg, Germany; 20 Division of Preventive Oncology, German Cancer Research Center (DKFZ) and National Center for Tumor Diseases (NCT), Heidelberg, Germany; 21 German Cancer Consortium (DKTK), German Cancer Research Center (DKFZ), Heidelberg, Germany; 22 Division of Cancer Medicine, University of Texas MD Anderson Cancer Center, Texas, TX USA; 23 Department of Epidemiology, Harvard T.H. Chan School of Public Health, Boston, MA USA; 24 Broad Institute of MIT and Harvard, Cambridge, MA USA; 25 Program in Molecular Pathological Epidemiology, Department of Pathology, Brigham and Women’s Hospital and Harvard Medical School, Boston, MA USA; 26 Cancer Immunology and Cancer Epidemiology Programs, Dana-Farber Harvard Cancer Center, Boston, MA USA; 27 Department of Inflammation Biology, School of Immunology and Microbial Sciences, King’s College London, London, UK

**Keywords:** Risk factors, Cancer

## Abstract

**Background:**

Traditional body-shape indices such as Waist Circumference (WC), Hip Circumference (HC), and Waist-to-Hip Ratio (WHR) are associated with colorectal cancer (CRC) risk, but are correlated with Body Mass Index (BMI), and adjustment for BMI introduces a strong correlation with height. Thus, new allometric indices have been developed, namely A Body Shape Index (ABSI), Hip Index (HI), and Waist-to-Hip Index (WHI), which are uncorrelated with weight and height; these have also been associated with CRC risk in observational studies, but information from Mendelian randomization (MR) studies is missing.

**Methods:**

We used two-sample MR to examine potential causal cancer site- and sex-specific associations of the genetically-predicted allometric body-shape indices with CRC risk, and compared them with BMI-adjusted traditional body-shape indices, and BMI. Data were obtained from UK Biobank and the GIANT consortium, and from GECCO, CORECT and CCFR consortia.

**Results:**

WHI was positively associated with CRC in men (OR per SD: 1.20, 95% CI: 1.03–1.39) and in women (1.15, 1.06–1.24), and similarly for colon and rectal cancer. ABSI was positively associated with colon and rectal cancer in men (1.27, 1.03–1.57; and 1.40, 1.10–1.77, respectively), and with colon cancer in women (1.20, 1.07–1.35). There was little evidence for association between HI and colon or rectal cancer. The BMI-adjusted WHR and HC showed similar associations to WHI and HI, whereas WC showed similar associations to ABSI only in women.

**Conclusions:**

This large MR study provides strong evidence for a potential causal positive association of the allometric indices ABSI and WHI with CRC in both sexes, thus establishing the association between abdominal fat and CRC without the limitations of the traditional waist size indices and independently of BMI. Among the BMI-adjusted traditional indices, WHR and HC provided equivalent associations with WHI and HI, while differences were observed between WC and ABSI.

## Introduction

Colorectal cancer (CRC) is one of the most common cancers in terms of incidence globally, ranking second and third in women and men, respectively, with an estimated 1.9 million incident cases worldwide in 2020 [1]. CRC is one of the leading obesity-related cancers [2]. The International Agency for Research on Cancer (IARC) estimated that in 2012 110,000 cases of CRC worldwide (85,000 of them in colon and 25,000 in rectum) were attributable to excess weight or obesity [3].

Obesity is a worldwide epidemic. According to the World Health Organization, over 650 million adults worldwide (11% of men and 15% of women) were affected by obesity in 2016, a three-fold increase since 1975 [4]. If these trends continue, by 2025 global obesity prevalence is expected to reach 18% in men and 21% in women [5]. The main anthropometric parameter used to evaluate general obesity is Body Mass Index (BMI), with BMI ≥ 30 kg/m^2^ being the cut-off for an adult to be considered with obesity. BMI has been positively associated in many observational studies with risk of CRC, especially with colon cancer and in men [6–9]. However, BMI is unable to distinguish between adipose tissue and muscular mass or between fat accumulation in different body compartments. This has motivated the use of additional anthropometric measurements, such as Waist Circumference (WC), Hip Circumference (HC), and their combination in the Waist-to-Hip Ratio (WHR), to better reflect body shape and adiposity accumulation patterns and to facilitate the discrimination between abdominal (assessed by WC and WHR) and gluteofemoral (i.e., around the hips, assessed by HC) fat accrual. Similarly to BMI, WC and WHR have also been associated with higher risk of CRC (especially colon cancer in men), while the available data for HC is inconclusive [9–11].

However, these traditional body shape indices are strongly positively correlated with BMI [12]. To overcome this limitation, few previous studies have performed adjustment of the traditional indices for BMI, with differing results ranging from no or small attenuation to not significant associations [10, 13]. An alternative approach implemented the development of new, BMI-independent allometric body shape indices, namely A Body Shape Index (ABSI), Hip Index (HI), and Waist-to-Hip Index (WHI). These indices are, in fact, mathematical transformations of WC, HC and WHR, respectively, normalized to height and weight; thus, they are uncorrelated with height and weight [14–16]. Among them, ABSI and WHI have been positively associated with colon and rectal cancer in men and with colon cancer in women, and an inverse association has been found between HI and colon cancer in men, albeit in limited studies [8, 17].

Mendelian randomization (MR) investigates the potential causal relationship between an exposure and an outcome with the use of genetic variants (usually single-nucleotide polymorphisms (SNPs)) as instrumental variables (IV); genetic variants are randomly allocated at meiosis and conception, thus their associations with disease outcomes are less vulnerable to environmental confounding or reverse causation bias. MR studies have positively associated genetically predicted BMI and WHR with CRC, but associations by subsite and sex were not fully investigated and HC was not included [18, 19]. No MR studies, to our knowledge, have examined the associations between the allometric indices and CRC risk.

Two-sample MR uses summary data from genome-wide association studies (GWAS). Previous GWAS have shown that the adjustment of WC and HC for BMI introduces a correlation with height, stronger than the association of the unadjusted WC and HC with height; [12] thus, the traditional indices, both non-BMI-adjusted and BMI-adjusted, present with a fundamental flaw: the non-BMI adjusted ones are correlated with BMI, and BMI-adjusted WC and HC are correlated with height, whereas the allometric indices are uncorrelated with height, weight and BMI by design. In addition, GWAS of BMI-adjusted WC and HC identify a larger number of SNPs associated with height compared to ABSI and HI [16]. This matters, because standing height has also been associated positively with CRC both in observational and MR studies, although the results of previous studies varied by sex and across tumor anatomical subsite [9, 20, 21]. It is unknown how these would influence the associations of BMI-adjusted WC and HC with CRC, compared to the associations of their allometric analogs ABSI and HI.

Our aim was to examine the associations of body shape with CRC risk using the allometric body-shape indices, and to compare these associations with those based on the traditional body shape indices and BMI, using an MR analysis. Height was also examined to assess whether an association between the traditional indices and height can explain any discrepancies between traditional and allometric indices. Furthermore, since results from previous studies have demonstrated that the association between obesity and CRC is stronger in men than women [22, 23], and considering the possible differential results across anatomical sites, we examined the associations for colon and rectal cancer, in men and women separately.

## Methods

The paper has been written in accordance with the Strengthening the Reporting of Observational Studies in Epidemiology using Mendelian Randomization (STROBE-MR) guidelines (Additional File 1) [24].

### Anthropometric GWAS data

**Fig. 1:**
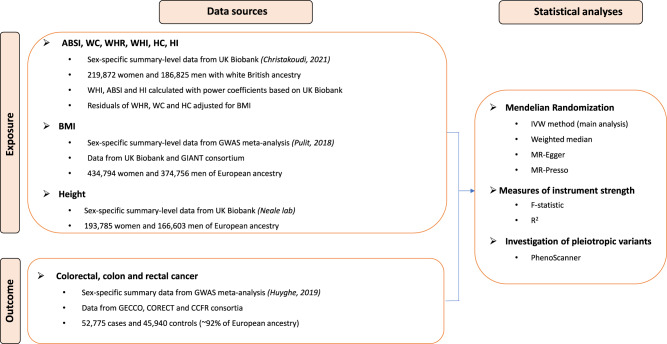
Analysis plan. ABSI A Body Shape Index, WC Waist Circumference, WHR Waist-to-Hip Ratio, WHI Waist-to-Hip Index, HC Hip Circumference, HI Hip Index, BMI Body Mass Index, GWAS Genome-Wide Association Study, GIANT Genetic Investigation of Anthropometric Traits, GECCO Genetics and Epidemiology of Colorectal Cancer Consortium, CORECT Colorectal Cancer Transdisciplinary Study, CCFR Colon Cancer Family Registry, IVW Inverse-Variance Weighted.

Sex-specific variants associated with WHI, ABSI, HI, WHR, WC, and HC were extracted from the summary statistics of a GWAS in 219,872 women and 186,825 men of European ancestry from the UK Biobank (Fig. 1) [16]. In this GWAS, WHR, WC and HC were adjusted for BMI in linear models, thus generating residuals; WHI, ABSI and HI were calculated using power coefficients derived from UK Biobank data, thus there is no residual correlation which may exist for the original coefficients for ABSI and HI, based on the National Health and Nutrition Examination Survey (NHANES) [14, 15]. Details regarding the genomic correlations between the allometric and traditional indices, as well as sex differences and the overlap between the genetic instruments in men and women for each allometric index, are provided in the respective publication [16]. Furthermore, all anthropometric indices were inverse normal transformed to a standard deviation (SD) scale with Blom’s method prior to examining associations with genetic polymorphisms [25].

For BMI, we extracted sex-specific variants from a meta-analysis of GWAS in 374,756 men and 434,794 women of European ancestry, with data from the UK Biobank (second release, June 2017) and the Genetic Investigation of Anthropometric Traits (GIANT) consortium [26], in which BMI was expressed in SD units. Sex-specific variants for standing height (standardized per SD) were obtained from a GWAS of 166,603 men and 193,785 women of European ancestry in the UK Biobank (Fig. 1) [27].

### Colorectal cancer GWAS data

The summary statistics for colorectal cancer (overall, site- and sex-specific) variants were retrieved from a recent GWAS meta-analysis, with data from three consortia: Genetics and Epidemiology of Colorectal Cancer Consortium (GECCO), Colorectal Cancer Transdisciplinary Study (CORECT) and the Colon Cancer Family Registry (CCFR) (Fig. 1) [28]. In these studies, men comprised 28,207 CRC cases and 22,204 controls and women comprised 24,568 CRC cases and 23,736 controls, with most of the participants (~92%) being of European ancestry. The UK Biobank contributed in this meta-analysis with 5356 cases (10.15% of all cases, 3081 men and 2275 women) and 21,407 controls (12,323 men and 9084 women).

### Statistical analysis

We performed MR to examine the association of each anthropometric index (WHI, ABSI, HI, WHR adjusted for BMI: WHRadjBMI, WC adjusted for BMI: WCadjBMI, HC adjusted for BMI: HCadjBMI, BMI, height), with (a) CRC overall, (b) colon, and (c) rectal cancer, using SNP estimates from sex-specific GWAS of exposures as well as outcomes.

Two-sample MR was used, since genetic associations for the exposure and the outcome had been estimated in different samples [29]. Genome-wide significant SNPs (i.e., with *p* < 5 × 10^−8^) were selected for each exposure. Exposure and outcome data were harmonized, to ensure that the effect allele was the same in the exposure and the outcome data, and inconsistent SNPs were removed (i.e., SNPs for which the effect allele of the exposure was different from either allele of the outcome data). Removal of SNPs that were in linkage disequilibrium was performed by clumping of the data (r^2^ < 0.001). For our main analyses, we used a random-effects inverse-variance weighted method (IVW).

The interpretation of MR is based on the following assumptions: (a) relevance (genetic variants are associated with the exposure), (b) independence (genetic variants are not associated with the confounders of the exposure and outcome), and (c) exclusion restriction (genetic variants do not affect the outcome except through the exposure) [30–33]. We used several methods to examine the validity of these assumptions. For the first assumption, we measured the strength of the genetic instruments, calculating the F-statistic and the proportion of the variance of the exposure explained (r^2^) by each SNP. To assess the second and third assumptions we searched the PhenoScanner database, which contains associations between SNPs and traits which were found in previous GWAS [34], to identify potential pleiotropic variants; analysis was repeated after excluding them. Weighted median, MR-Egger and MR-Presso methods were also performed to further assess the robustness of our findings. In the weighted median method, the contribution of each genetic variant is proportional to its weight, and the estimate provided is consistent if less than 50% of the weight comes from invalid IVs [35]. The intercept of the MR-Egger method indicates potential pleiotropy, and the slope provides an estimate of the causal effect, which is consistent even when all instrumental variables are invalid. The MR-Egger method assumes that the direct pleiotropic effects of the genetic variants on the outcome are distributed independently of the genetic associations with the exposure, known as the Instrument Strength Independent of Direct Effect (InSIDE) assumption [36]. The MR-Presso method detects outlying variants and repeats the analysis without them [37].

In an additional sensitivity analysis, for each traditional index we calculated the percentage of SNPs which were overlapping or were strongly correlated (r^2^ ≥ 0.8) with the SNPs of its allometric analog. Subsequently, we split the SNPs of each traditional index into two groups: the shared ones (i.e., the SNPs which fulfilled the aforementioned criteria), and those which were unique for the traditional index (i.e., not overlapping and not strongly correlated (r^2^ < 0.8) with the SNPs of its allometric analog); the analysis was repeated for each of these sets of SNPs.

Furthermore, to examine the differences between the genetic instruments in men and those in women, we calculated the percentage of SNPs that were overlapping or strongly correlated (r^2^ ≥ 0.8) between men and women for each allometric index.

MR estimates represent the change in outcome per SD change in the genetically predicted exposure. Estimates for CRC, colon and rectal cancer reflect odds ratios (OR). The statistical analyses were performed using R, version 4.0.3 and the Mendelian randomization R package (version 0.6.0) [38].

## Results

### Associations of body shape with CRC

**Fig. 2:**
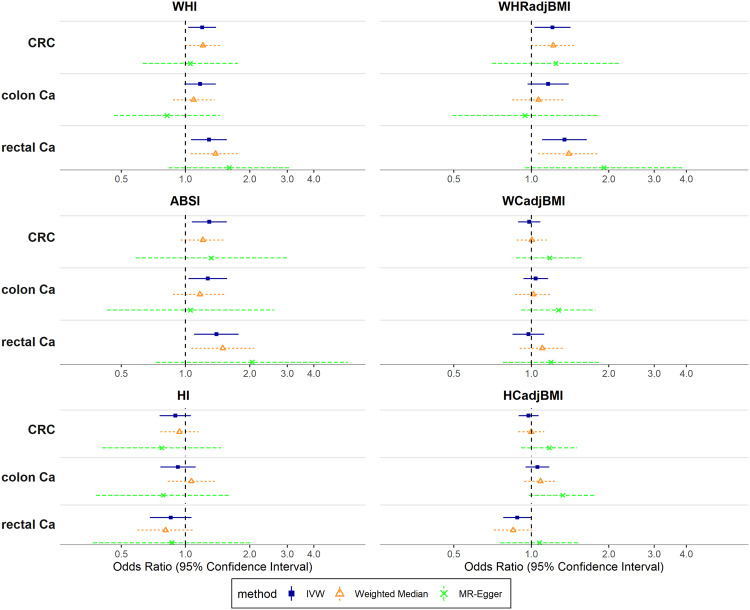
Associations of allometric and traditional body shape indices with colorectal, colon and rectal cancer (men). Odds Ratios (OR) reflect the change in outcome risk per standard deviation change in the genetically predicted index. CRC colorectal cancer, Ca cancer, WHI Waist-to-Hip Index, WHRadjBMI Waist-to-Hip Ratio adjusted for Body Mass Index, ABSI A Body Shape Index, WCadjBMI Waist Circumference adjusted for Body Mass Index, HI Hip Index, HCadjBMI Hip Circumference adjusted for Body Mass Index, IVW Inverse-Variance Weighted.

In men, using IVW, genetically predicted WHI was associated positively with CRC risk overall (OR_IVW_:_:_ 1.20; 95% confidence interval [CI]: 1.03–1.39 per one SD), similarly for colon (OR_IVW_: 1.17; 95% CI: 0.99–1.39) and rectal cancer (OR_IVW_: 1.29; 95% CI: 1.06–1.56). A positive association was also found between ABSI and CRC risk overall (OR_IVW_: 1.29; 95% CI: 1.07–1.56), similarly for colon (OR_IVW_: 1.27; 95% CI: 1.03–1.57) and rectal cancer (OR_IVW_: 1.40; 95% CI: 1.10–1.77). There was little evidence for associations of HI with CRC risk, except for a potential suggestive association with rectal cancer (OR_IVW_: 0.85; 95% CI: 0.68–1.07) (Fig. 2). Estimates from weighted median were broadly in agreement with IVW, and there was little evidence of between SNP heterogeneity (all p_intercept,MR-Egger_ > 0.10) (Fig. 2, Supplementary Table 1 in Additional File 2).

Also in men, the associations of WHRadjBMI with CRC risk overall and individually with colon and rectal cancer resembled the associations with WHI. There was little evidence, however, for positive associations with WCadjBMI and any outcome. HCadjBMI appeared associated inversely with rectal cancer risk (OR_IVW_: 0.88; 95% CI: 0.78–1.00), but not with other outcomes (Fig. 2, Supplementary Table 1 in Additional File 2).

**Fig. 3:**
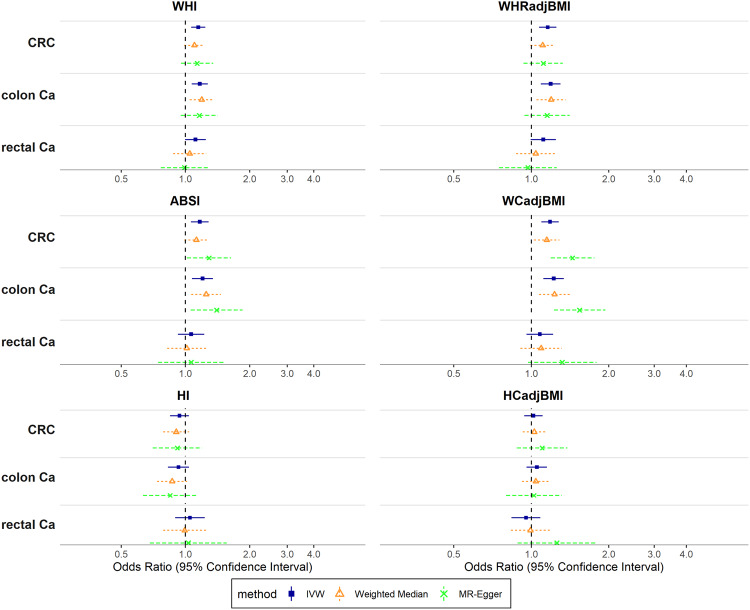
Associations of allometric and traditional body shape indices with colorectal, colon and rectal cancer (women). Odds Ratios (OR) reflect the change in outcome risk per standard deviation change in the genetically predicted index. CRC colorectal cancer, Ca cancer, WHI Waist-to-Hip Index, WHRadjBMI Waist-to-Hip Ratio adjusted for Body Mass Index, ABSI A Body Shape Index, WCadjBMI Waist Circumference adjusted for Body Mass Index, HI Hip Index, HCadjBMI Hip Circumference adjusted for Body Mass Index, IVW Inverse-Variance Weighted.

In women, using IVW, WHI was associated positively with CRC risk overall (OR_IVW_: 1.15; 95% CI: 1.06–1.24), similarly for colon (OR_IVW_: 1.17; 95% CI: 1.07–1.27) and rectal cancer (OR_IVW_: 1.11; 95% CI: 0.99–1.24). ABSI was also associated positively with CRC risk overall (OR_IVW_: 1.17; 95% CI: 1.06–1.28), but individually only with colon cancer (OR_IVW_: 1.20; 95% CI: 1.07–1.35). There was little evidence for associations of HI with CRC risk overall, or individually with colon and rectal cancer (Fig. 3). Estimates for allometric indices from both weighted median and MR-Egger were broadly in agreement with IVW. Estimates for traditional indices were mainly consistent with those for allometric indices (Fig. 3, Supplementary Table 1 in Additional File 2).

### Associations of body size with CRC

**Fig. 4:**
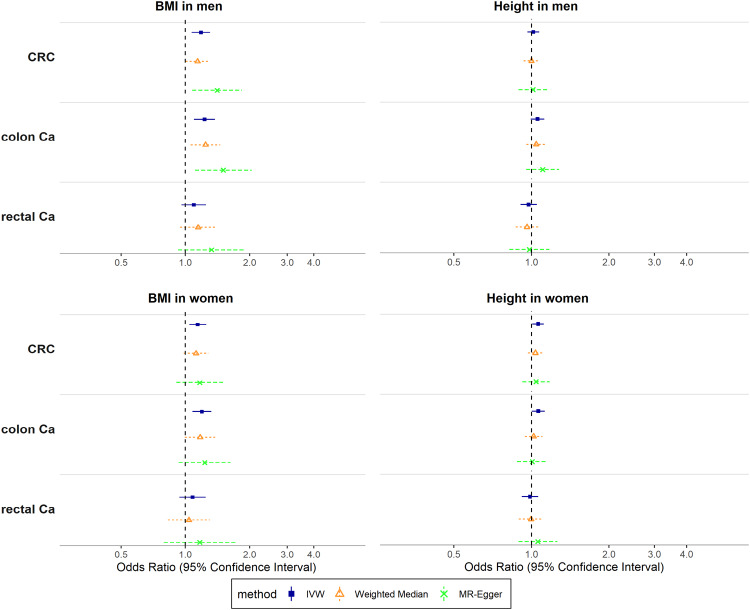
Associations of Body Mass Index and height with colorectal, colon and rectal cancer (both sexes). Odds Ratios (OR) reflect the change in outcome risk per standard deviation change in the genetically predicted index. CRC colorectal cancer, Ca cancer, BMI Body Mass Index, IVW Inverse-Variance Weighted.

In both men and women, genetically predicted BMI was associated positively with CRC risk overall (OR_IVW_: 1.18; 95% CI: 1.07–1.30 for men, OR_IVW_: 1.14; 95% CI: 1.04–1.25 for women), but separately only for colon cancer (OR_IVW_: 1.23; 95% CI: 1.10–1.38 for men, OR_IVW_: 1.19; 95% CI: 1.08–1.32 for women) and not for rectal cancer. Height showed weak positive associations with colon cancer risk (OR_IVW_: 1.06; 95% CI: 0.99–1.12 for men, OR_IVW_: 1.06; 95% CI: 1.00–1.13 for women) (Fig. 4). Estimates for weighted median and MR-Egger were mainly compatible with estimates from IVW for BMI, but provided little evidence for associations with height (Fig. 4, Supplementary Table 1 in Additional File 2).

### Evaluation of the MR assumptions, secondary and sensitivity analyses

In our main analysis, no evidence for weak instrument was found, as the F-statistics for all SNPs were >10, ranging from 26 to 1213 (Supplementary Tables 2, 3 in Additional File 2). Secondary analyses using MR-Presso method provided similar results with the main analysis (Supplementary Table 1 in Additional File 2).

In sensitivity analysis, we searched the PhenoScanner database for potential pleiotropic genetic variants. We found 43 SNPs in total that were also associated with type 2 diabetes mellitus or glycated hemoglobin; 28 SNPs that were in common with alcohol intake; 19 SNPs that were in common with inflammatory bowel disease (IBD); and 10 SNPs which were also associated with smoking (Supplementary Table 4 in Additional File 2). Further analysis after excluding each one of these sets of SNPs provided similar results (Supplementary Table 5 in Additional File 2).

In another sensitivity analysis, for each traditional index we calculated the percentage of SNPs that overlapped or were strongly correlated with the SNPs of its allometric analog; it ranged from 12.79% (for HCadjBMI in men) to 92.77% (for WHRadjBMI in women), with a median value of 33.77% (Supplementary Table 6 in Additional File 2). Separate analysis of the shared and the unique SNPs of each traditional index showed that, when the unique SNPs were removed, WCadjBMI, similarly to ABSI, was associated in a positive direction with CRC, colon and rectal cancer risk in men (29 SNPs shared with ABSI, 20% of all), although without a nominal statistical significance. The associations of WCadjBMI in women and HCadjBMI, WHRadjBMI in both sexes using the unique (except when the unique SNPs were too few [i.e., 7] for WHRadjBMI in men) and the shared SNPs were like those of the initial analysis (Supplementary Table 6 in Additional File 2). Of note, the suggestive inverse association of HCadjBMI with rectal cancer risk in men appeared stronger for the 28 SNPs shared with HI (13% of all) than for the unique SNPs.

Furthermore, for each allometric index we calculated the percentage of SNPs that overlapped or were strongly correlated between men and women. It ranged from 5,65% (for ABSI in women) to 25% (for WHI in men) (Supplementary Table 7 in Additional File 2).

## Discussion

This is the first MR study that has examined the association of the allometric body-shape indices with CRC risk overall and by subsite, separately in men and women. Genetically predicted WHI was positively associated with CRC risk similarly by subsite and sex. ABSI showed positive associations with colon and rectal cancer risk in men and with colon cancer risk in women. Little evidence was found for an association between HI and CRC risk, except for a suggestive inverse association with rectal cancer risk in men.

Data from previous studies regarding the associations of the new allometric indices with CRC risk is limited. The MR associations we found for ABSI and WHI are consistent with the observational associations previously reported in the UK Biobank, although the association of WHI with colon cancer risk in men was stronger in the observational study [17]. Results from other observational studies regarding ABSI vary, however these studies had small sample sizes [8], or they performed site-combined analyses [39, 40]. Regarding HI, we have shown previously an inverse association between HI and colon cancer risk in men in UK Biobank [17]. In general, our findings corroborate the limited literature data regarding WHI and ABSI, whereas for HI there is discordance; an inverse association was found in men in both the observational and MR studies, albeit in different location (colon vs. rectal cancer) and our MR finding was not nominally significant.

Previous observational studies have shown positive associations of the non-BMI-adjusted traditional indices WHR and WC with CRC risk [9, 11, 17], and positive or null for the non-BMI-adjusted HC [13, 41, 42]. When WHR and WC were adjusted for BMI, the results varied from no or small attenuation of the positive association to not significant associations [8, 10, 13, 17, 41]. For HC, the associations were not significant after adjustment for BMI [13].

Data comparing the associations of the allometric and traditional indices with CRC risk in the same sample are scarce, and partially agree with our work; we have previously found in the UK Biobank similar associations between WHI and WHR and between ABSI and WC (for BMI-adjusted and non-BMI-adjusted WHR and WC), and between HI and HC (only after adjustment of HC for BMI) [17].

The biology underlying these associations is complex. Adipose tissue is not a homogeneous entity, as deposits of fat in distinctive parts of the body have differing characteristics and metabolic functions. Visceral adipose tissue is pro-inflammatory with accumulation of cytokines and other inflammatory mediators and is strongly associated with insulin resistance, whereas gluteofemoral adipose tissue has a lower pro-inflammatory profile, is positively associated with insulin sensitivity and protects from ectopic fat accumulation [43–47]. The biological pathways linking adipose tissue and cancer, involve, among others, insulin resistance, adipose-tissue derived inflammation with secretion of proinflammatory cytokines, and sex hormones metabolism [48–51]. Thus, these inherent differences between visceral and gluteofemoral fat deposits, apart from the hazardous and protective effects they have, respectively, on diabetes and cardiovascular diseases which have been acknowledged in numerous studies [52–57], might also explain their differing role on cancer development; visceral fat seems to enhance the activity of cancer-related pathways, whereas gluteofemoral fat shows a possible protective role. Furthermore, hip size does not reflect only the accumulation of gluteofemoral adipose tissue, but it might be increased by greater volume of gluteal muscles and bone structure, which in turn is a possible indication of increased physical activity, an acknowledged protective factor regarding CRC. Our findings corroborate the theoretical knowledge linking visceral fat and CRC: we have found significant positive associations between the allometric indices of waist size (WHI and ABSI) and CRC risk, thus, we have positively linked abdominal adiposity and CRC, without the limitations of the traditional waist size indices and BMI. Regarding the potential protective role gluteofemoral fat has on CRC, our results are not conclusive, as the suggestive inverse association we found between HI and rectal cancer risk in men was not nominally significant.

Regarding body-size and body shape indices, WC, HC, height and weight are primary traits, which means that they can and have been measured. On the contrary, BMI and the allometric body shape indices are mathematical transformations, which do not exist in nature and have been created to serve a purpose. WHI, ABSI, and HI reflect body shape among individuals with the same weight and height and they are uncorrelated with weight and height, that is, they relate body shape to the primary traits; they are unbiased estimators of body shape. The adjustment of the traditional indices for BMI aims to achieve the same but relates body shape to a mathematical transformation of weight and height, which introduces a completely artificial stronger positive association of WCadjBMI and HCadjBMI with height, resulting in biased indices of body size. The discrepancies we found between WCadjBMI and its allometric analog ABSI, reinforce this theoretical knowledge. WHRadjBMI is also theoretically incorrect but since it is uncorrelated with height, shows similar associations to WHI. Regarding HCadjBMI, although this does not differ materially from HI with respect to the associations with CRC, it remains a theoretically incorrect index, which may potentially show bias with respect to other outcomes.

This study has several strengths. To the best of our knowledge, this is the first study to examine the MR associations of body-shape with CRC independently of overall body size reflected in BMI and height, using the allometric body shape indices. This is also the first study to compare allometric with traditional body shape indices in an MR analysis. An additional strength is the large sample size used and the possibility to perform site- and sex-specific analyses. The MR method, by its design, enabled us to examine these associations minimizing the possibility of reverse causation bias and excluding the effect of several potential confounders. Finally, we performed secondary analyses to examine the MR assumptions.

However, there are some limitations. We used data from a European ancestry population, therefore our findings may not be generalizable to other ethnicities. The two-sample MR approach operates under the assumption of a linear association between the exposure and the outcome, thus the possibility of non-linear associations could not be examined. However, a previous meta-analysis has reported just a slight deviation from linearity with a steeper association above 27 kg/m^2^ [58]. Furthermore, the GWAS datasets for the exposures and outcomes were partly overlapping, which may have introduced bias, but it has been shown that such bias is unlikely to be considerable when genetic instruments are strong [59]. Further CRC subtypes regarding histological and molecular characteristics are currently lacking from relevant GWAS, thus additional analyses could not be pursued. Finally, the MR approach uses as exposure the genetic tendency for higher adiposity indices measured at a single point in time; however, obesity may undergo changes over an individual’s lifetime, and a recent case-control study showed that cumulative lifetime exposure to excess weight is probably a much stronger predictor of CRC risk than measures at a single time point [60].

In conclusion, we found evidence of a positive association between the allometric indices of waist size and CRC risk, which were similar by tumor subsite and sex. Thus, abdominal adiposity is positively associated with CRC risk, independently of overall adiposity and without the limitations of the traditional waist size indices. Hip size, evaluated allometrically by HI, was not associated with CRC, except for a suggestive inverse association with rectal cancer risk in men that requires further investigation. Regarding the traditional indices, while the associations of WHRadjBMI and HCadjBMI were similar to their allometric equivalents, those of WCadjBMI were biased towards the null in men. If traditional approaches are preferred, the residuals should be derived from models adjusting for weight and height and not for BMI.

### Supplementary information

Additional File 1

Additional File 2

## Data Availability

All data used in this work are presented in the Additional files that accompany the manuscript and are available in the original publications. The summary statistics for allometric body shape indices are available from the NHGRI-EBI GWAS Catalog at https://www.ebi.ac.uk/gwas/publications/34021172.
